# Longitudinal assessment of quality assurance measurements in a 1.5T MR‐linac: Part I—Linear accelerator

**DOI:** 10.1002/acm2.13418

**Published:** 2021-09-10

**Authors:** Ergys Subashi, Seng Boh Lim, Xesus Gonzalez, Neelam Tyagi

**Affiliations:** ^1^ Department of Medical Physics Memorial Sloan Kettering Cancer Center New York New York; ^2^ Elekta Limited Stockholm Sweden

**Keywords:** longitudinal, MR‐linac, 1.5T, quality assurance

## Abstract

**Purpose:**

To describe and report longitudinal quality assurance (QA) measurements for the mechanical and dosimetric performance of an Elekta Unity MR‐linac during the first year of clinical use in our institution.

**Materials and methods:**

The mechanical and dosimetric performance of the MR‐linac was evaluated with daily, weekly, monthly, and annual QA testing. The measurements monitor the size of the radiation isocenter, the MR‐to‐MV isocenter concordance, MLC and jaw position, the accuracy and reproducibility of step‐and‐shoot delivery, radiation output and beam profile constancy, and patient‐specific QA for the first 50 treatments in our institution. Results from end‐to‐end QA using anthropomorphic phantoms are also included as a reference for baseline comparisons. Measurements were performed in water or water‐equivalent plastic using ion chambers of various sizes, an ion chamber array, MR‐compatible 2D/3D diode array, portal imager, MRI, and radiochromic film.

**Results:**

The diameter of the radiation isocenter and the distance between the MR/MV isocenters was (*μ* ± *σ*) 0.39 ± 0.01 mm and 0.89 ± 0.05 mm, respectively. Trend analysis shows both measurements to be well within the tolerance of 1.0 mm. MLC and jaw positional accuracy was within 1.0 mm while the dosimetric performance of step‐and‐shoot delivery was within 2.0%, irrespective of gantry angle. Radiation output and beam profile constancy were within 2.0% and 1.0%, respectively. End‐to‐end testing performed with ion‐chamber and radiochromic film showed excellent agreement with treatment plan. Patient‐specific QA using a 3D diode array identified gantry angles with low‐pass rates allowing for improvements in plan quality after necessary adjustments.

**Conclusion:**

The MR‐linac operates within the guidelines of current recommendations for linear accelerator performance, stability, and safety. The analysis of the data supports the recently published guidance in establishing clinically acceptable tolerance levels for relative and absolute measurements.

## 1 INTRODUCTION

MR‐guided systems, particularly the integrated MR‐linac,^1^, ^2^, ^3^, ^4^ provide a novel platform for the delivery of precision radiotherapy and may enable improvements in therapeutic response by increasing dose to the target while sparing organs at risk.^5^, ^6^, ^7^, ^8^ The clinical implementation of these systems necessitates a review of commissioning and quality assurance (QA) methods and, if needed, revisions to address differences with conventional radiotherapy machines. Integrated MR‐linacs present with challenges that are not encountered when each component is considered separately. Furthermore, these devices allow for online plan adaptation strategies ranging from simple dose calculation to inverse planning for intensity modulated radiation therapy (IMRT). Online adaptation is comprised of numerous steps including acquisition of daily planning MRI images, rigid and deformable registration, contouring, IMRT optimization, and dose calculation in the presence of the magnetic field.^9^, ^10^ Commissioning and QA methods for each of these steps are described in several national and international reports on conventional linacs and MRI‐simulators.^11^, ^12^, ^13^, ^14^, ^15^, ^16^

The Elekta Unity MR‐linac (Elekta AB, Stockholm, Sweden) was cleared by the US Food and Drug Administration (FDA) for clinical use in the United States in late December 2018. This device couples a diagnostic 1.5T MRI scanner (Philips Healthcare, Best, the Netherlands) with a linear accelerator equipped with a 7 MV flattening filter‐free treatment beam. Dose calculation and optimization is implemented in a Monte Carlo‐based treatment planning system (TPS) that is able to model the effect of the magnetic field.^17^, ^18^ This TPS is employed offline to generate a reference plan to be used for adaptation, and online for optimization and dose calculation in the daily adapted plan. Online plan adaptation is achieved in two ways, broadly based on user workflows that allow for a virtual couch shift and no contour editing (adapt‐to‐position, ATP) or full replanning with daily contouring (adapt‐to‐shape, ATS).^9^

Multiple studies have described methods, equipment, and recommendations for commissioning and quality assurance in the integrated MR‐linac.^19^, ^20^, ^21^, ^22^, ^23^, ^24^ While national and international associations have specified recommendations and tolerances for commissioning and quality assurance in conventional machines, currently there are no published consensus protocols specific for MR‐linacs. A consortium of early clinical users, developers, and manufacturers has recently published recommendations for quality assurance for the Elekta Unity system.^25^ The longitudinal assessment of QA measurements provides additional necessary information about machine performance, stability, and safety. The analysis of this data offers further guidance in establishing clinically acceptable tolerance levels for relative and absolute measurements. In this work, we report the 1‐year longitudinal trend of relevant mechanical and dosimetric linac QA measurements for an Elekta Unity machine during clinical use in our institution. We also report baseline measurements from end‐to‐end QA performed using anthropomorphic phantoms with ion chamber and film. The analysis of the QA measurements for the MRI component of Unity will be described in a forthcoming publication.

## 2 MATERIALS AND METHODS

### 2.1 Radiation isocenter

**FIGURE 1:**
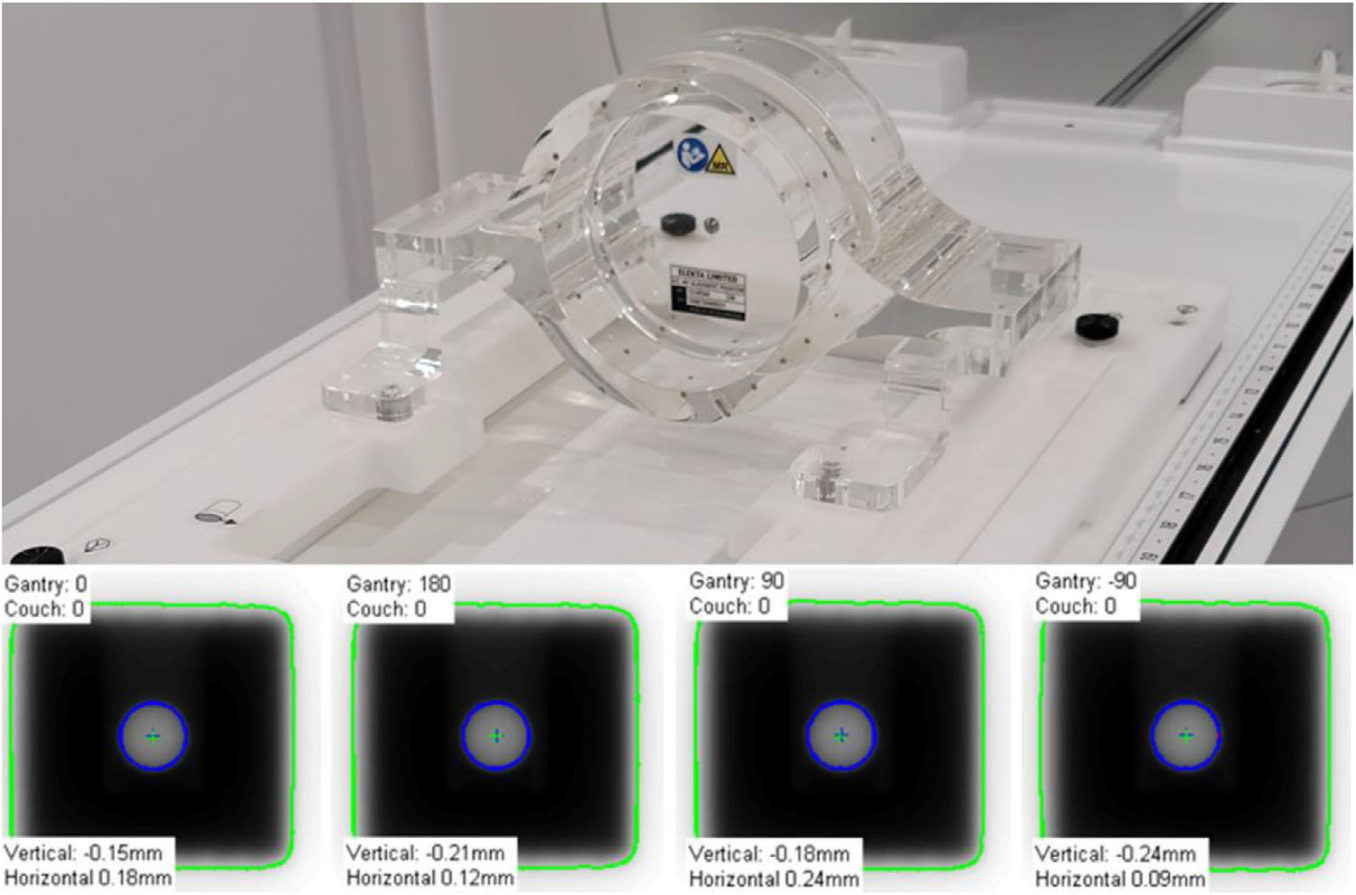
(Top) Setup for Winston–Lutz test performed using a vendor provided MV‐phantom immobilized in the QA platform. (Bottom) Example of Central BB irradiated using a 3 × 3 cm^2^ field size at four cardinal gantry angles and captured on the MV panel

The size of the radiation isocenter was determined using a Winston–Lutz test. A vendor‐provided MV phantom, shown in Figure 1, combined with a QA platform,^25^ allowed for positioning at isocenter a central ball‐bearing (BB) with known dimensions. The relative change in position of the central BB with respect to the edges of the known radiation field is a measure of the size of the radiation isocenter. This is quantified by reporting the diameter of a sphere encompassing the position of the central axis over the measured gantry angles.^26^, ^27^ MV images at twelve equidistant gantry angles were acquired and analyzed using an in‐house developed program and cross‐checked with the RIT Isocenter Analysis Tool (Radiological Imaging Technology, Colorado Springs, CO). Gantry angles over attenuating structures in the cryostat, treatment couch, or QA platform were excluded from the analysis if field‐edge detection was affected. The tolerance used for the test that determines the size of the radiation isocenter was 1.0 mm in diameter.

### 2.2 MR‐to‐MV concordance

**FIGURE 2:**
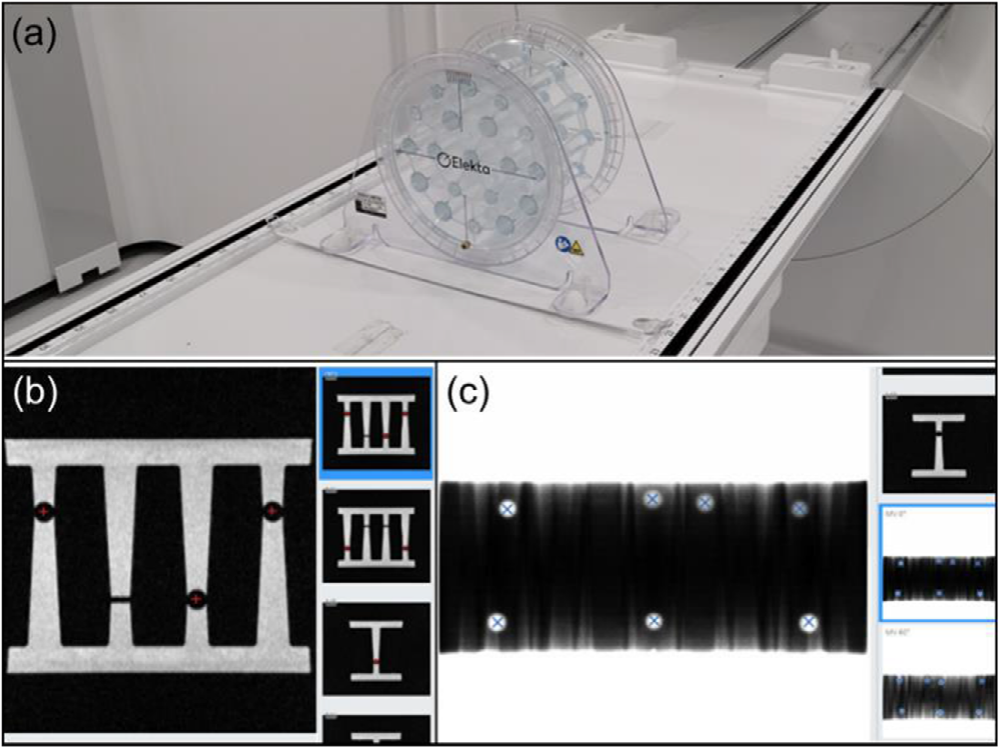
(a) Setup for MR‐to‐MV isocenter concordance test using a vendor provided phantom. (b) Example of MRI image showing location of automatically detected spheres (red plus signs). (c) Example of MV image showing location of automatically detected spheres (blue cross signs)

The MR‐to‐MV test measures the distance between the coordinates of the MRI and radiation isocenters. This test is performed with a vendor phantom, shown in Figure 2, containing seven ceramic spheres in a known geometry. The spheres, immersed in copper sulfate, appear opaque in MV images and create signal voids in MRI. The center of each sphere is detected in both modalities and a comparison of their relative positions provides a measure for MR‐to‐MV concordance. Ten MV images at gantry angles 0°, 60°, 78°, 102°, 117°, 180°, 240°, 258°, 282°, and 300° are acquired simultaneously with a T1‐weighted MRI image and analyzed using a vendor‐provided software package. At our institution, this test is performed daily by radiation therapists and monthly by a medical physicist. The tolerance used in the test that determines the MR‐to‐MV concordance was 1.0 mm with respect to baseline acquired at commissioning or after machine service.

### 2.3 MLC and jaw position

**FIGURE 3:**
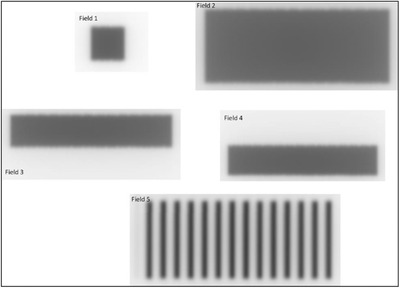
Example of MV images from vendor‐provided QA plan used in the test of MLC and jaw positional accuracy

Due to machine geometry and location of portal imager (265 cm from target), the maximum imaging FOV available for QA (21.0 × 8.5 cm^2^) in the MV panel cannot fit all MLC leaves. For this reason, during commissioning, multiple adjacent EBT3 radiochromic films were used to characterize the accuracy of leaf positioning. The central 28 leaves and the jaws were then monitored monthly using a vendor provided radiation plan that includes multiple field sizes and shapes, as shown in Figure 3. The acquired images are analyzed using a vendor‐provided software package that is able to calculate the position of MLC leaves set at Y (Sup‐Inf) = 0, 40 mm and jaws set at X (Lt‐Rt) = 100 mm. The output of the analysis is used to compute the root‐mean‐square (RMS) error over several field sizes and gantry angles. The tolerance used in the test that determines the MLC and jaw positional accuracy was 1.0 mm for RMS error.

### 2.4 Dosimetry of dynamic MLC delivery

In the Unity MR‐linac, the MLC round edge transmission is implicitly incorporated in the MLC position in the treatment planning system and is not customizable. The combination of the MLC transmission, mechanical integrity, and positional accuracy has an impact on the dosimetric accuracy of an IMRT delivery.^28^, ^29^ To quantify the accuracy and stability of the MLC model in the TPS, the A12MR ion chamber was inserted in water‐equivalent plastic and placed at isocenter at depth of 5.0 cm to measure the delivery of five sequences of 0, 5, 6, 8, 10 mm MLC apertures traveling from –5.0 cm to 5.0 cm. The delivery was in step‐and‐shoot mode using a total of 100 monitor units (MU). The apertures were adjacent to each other, traveled in the in‐out direction, and delivered an equal number of monitor units. The measurements were normalized to the output of an open field of 10.0 × 10.0 cm^2^ with the same MUs to generate a dynamic MLC (dMLC) relative dose. The measured relative dose was compared with the relative dose computed in the treatment planning system. The calculation in the TPS was performed using a 2.0 mm grid size and 0.5% statistical uncertainty. The QA test is performed monthly, and the tolerance was set to 3% change from the baseline measured during commissioning.

### 2.5 Output constancy

There are currently no published protocols for reference dosimetry in an MR‐linac. Early users of the Elekta MR‐linac have implemented IAEA TRS‐398^30^ and the formalism presented by O'Brien et al.^31^ for absolute dose calibration. TRS‐398 is used mainly due to the large SAD of the machine and the associated difficulty in measuring or calculating the beam quality specifier required for AAPM‐TG51. The protocol adopts TPR_20/10_ as the beam quality specifier, which in our machine was found to be TPR_20/10 _= 0.708. In our institution we adopted the TRS‐398 protocol and calibrated the machine to have an output of 1 cGy/MU at SAD, gantry 90°, depth of 5.0 cm (measured at 10.0 cm), for a 10.0 × 10.0 cm^2^ field size. The measurements were performed in water using the A12MR Farmer‐type chamber (Standard Imaging, Middleton, WI) aligned parallel to the magnetic field. The magnetic field conversion factor, *k_B_*, was calculated by Malkov and Rogers.^32^ The choice for calibrating at 90° is to minimize the potential impact that changes in the helium level would have in overall output. The output was independently confirmed by thermoluminescent dosimeters provided by the Imaging and Radiation Oncology Core at MD Anderson. We currently monitor output daily with the MR‐compatible DQA device (DQA3‐MR, Sun Nuclear Corporation, Melbourne, FL). For a period of 2 months, daily output was also monitored with the MV panel at G0 and G90 and cross‐referenced with weekly output measurements with the IC Profiler (Sun Nuclear Corporation, Melbourne, FL). The method and software for output measurements with the MV panel are provided by the vendor and have been described elsewhere.^25^, ^33^ Monthly output was determined using the formalism in IAEA TRS‐483.^34^ The chamber insert in the water‐equivalent plastic was filled with water in order to minimize the presence of air pockets. The tolerances used in the tests that monitor output were 3% for daily QA and 2% for weekly or monthly QA.

### 2.6 Beam profile constancy

Beam flatness and symmetry were monitored monthly with the IC Profiler using an open field of size 22.0 × 22.0 cm^2^. Flatness and symmetry were calculated over the central 80% of the field using the variance and point ratio, respectively. The calculated values were cross‐referenced with the treatment planning system. The tolerance used in the test that monitors profile beam constancy was 1% with respect to baseline measured during commissioning.

### 2.7 End‐to‐end QA

End‐to‐end testing was performed with the Quasar MRI4D phantom (Modus Medical Devices, London, Canada) and multimodality StereoPhan phantom (Sun Nuclear Corporation, Melbourne, FL). Each phantom was scanned in a CT‐simulator using a site‐specific protocol followed by the standard clinical planning and delivery procedures.

The Quasar phantom consists of an outer oval shell, two cylindrical compartments filled with water, and an air‐filled spherical target. An insert for an ionization chamber was filled with water to minimize the magnetic‐filed induced effects due to the presence of air bubbles, and A28MR (Standard Imaging, Middleton, WI) was used to measure charge. Charge was converted to dose based on an open field of 10.0 × 10.0 cm^2^ calibration and compared with the dose calculated in the treatment planning system. TPS dose in the ion‐chamber was estimated as the average of voxels inside the active volume (15 voxels). A 5‐field IMRT plan was developed in the TPS with a prescription of 15 Gy in a single fraction. All relevant structures were contoured and assigned bulk electron densities based on the mean Hounsfield units from the planning CT scan. Dose calculation was performed using a 2.0 mm grid size and 1% statistical uncertainty. The treatment plan was delivered twice after adaptation either with the method of adapt‐to‐position (ATP) with optimized weights or adapt‐to‐shape (ATS) with optimized weights/shapes from fluence.

The StereoPhan phantom consists of a cube filled with an MR liquid that allows for online image registration and a film insert for radiochromic film. The film insert has five MR‐safe titanium fiducials and orientation marks to assist in registration with the planning CT and evaluate localization uncertainties. The exposed films were scanned with an EPSON 12000XL scanner (Los Alamitos, CA) at 150 dpi and calibrated with FilmQA Pro (Ashland, Bridgewater, NJ) using the single‐scan protocol.^35^ The calibrated films were analyzed with in‐house software to determine the dosimetric and localization accuracy. A 5‐field IMRT plan was developed in the TPS with a prescription of 15 Gy in a single fraction. Dose calculation was performed using a 2.0 mm grid size and 1% statistical uncertainty. The plan was delivered after ATS adaptation with radiochromic films in both axial and sagittal orientation. An OD‐to‐ED calibration curve was obtained at standard calibration conditions using an open field of 10.0 × 10.0 cm^2^, 5 cm depth, and a range of 400–2200 monitor units. The films were analyzed for both dosimetric and localization accuracy.

**FIGURE 4:**
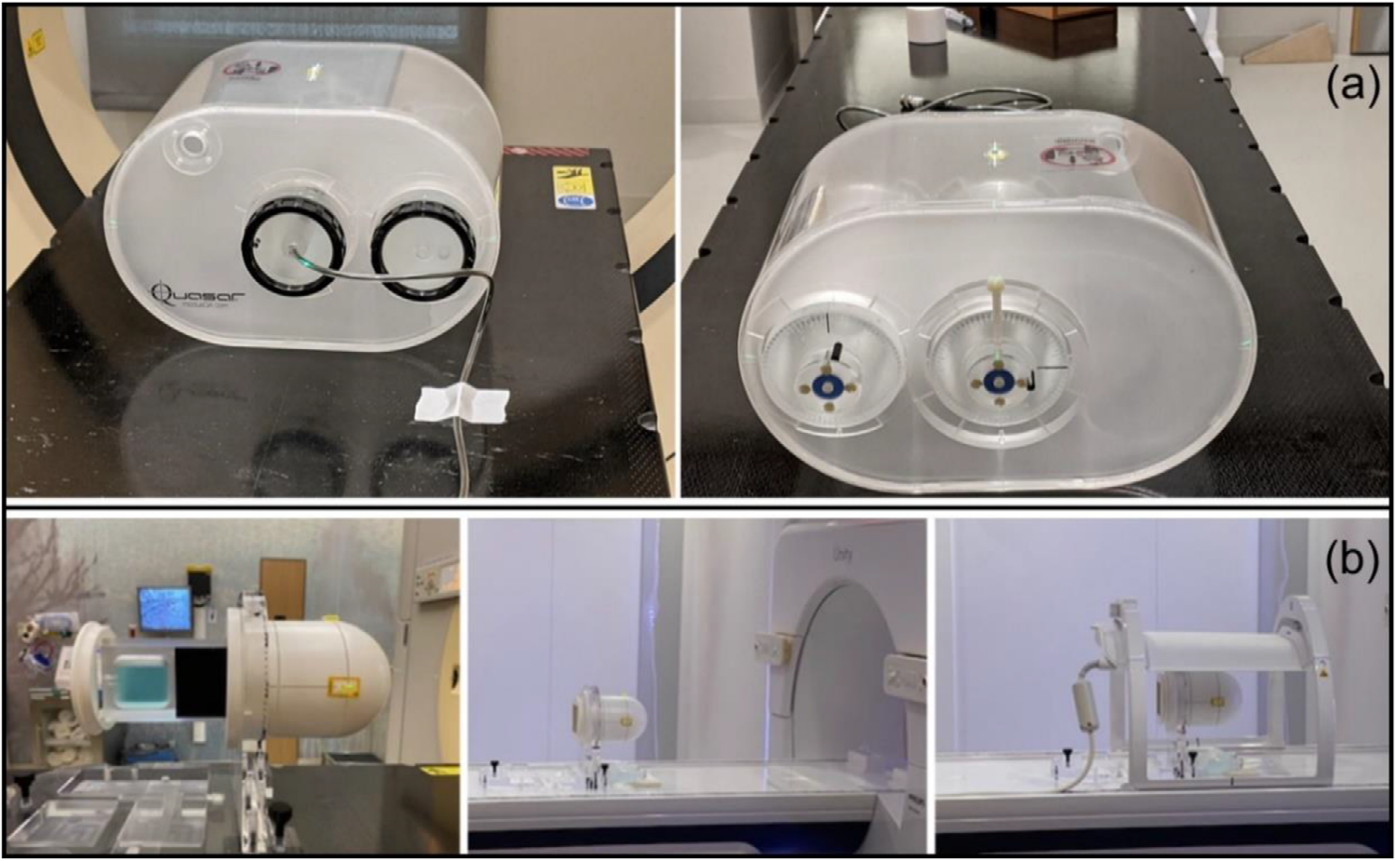
(a) Setup for end‐to‐end testing using the A28MR ion‐chamber and the Quasar MRI4D phantom. ATP/ATS plans were delivered using this geometry and the measured point‐dose was compared to TPS dose. (b) Setup for end‐to‐end testing using radiochromic film and the StereoPhan phantom. ATS plans were delivered using this geometry and the measured dose distribution was compared to TPS

Figure 4 shows the setup for both phantoms used for end‐to‐end testing. The tolerance used in the end‐to‐end tests was 2% for point dose measurements with the ion‐chamber and 5%/1.0 mm for dose‐difference/localization accuracy in dose distribution measurements with radiochromic film.^15^, ^36^

### 2.8 Patient‐specific QA using MR‐compatible 3D diode array

**FIGURE 5:**
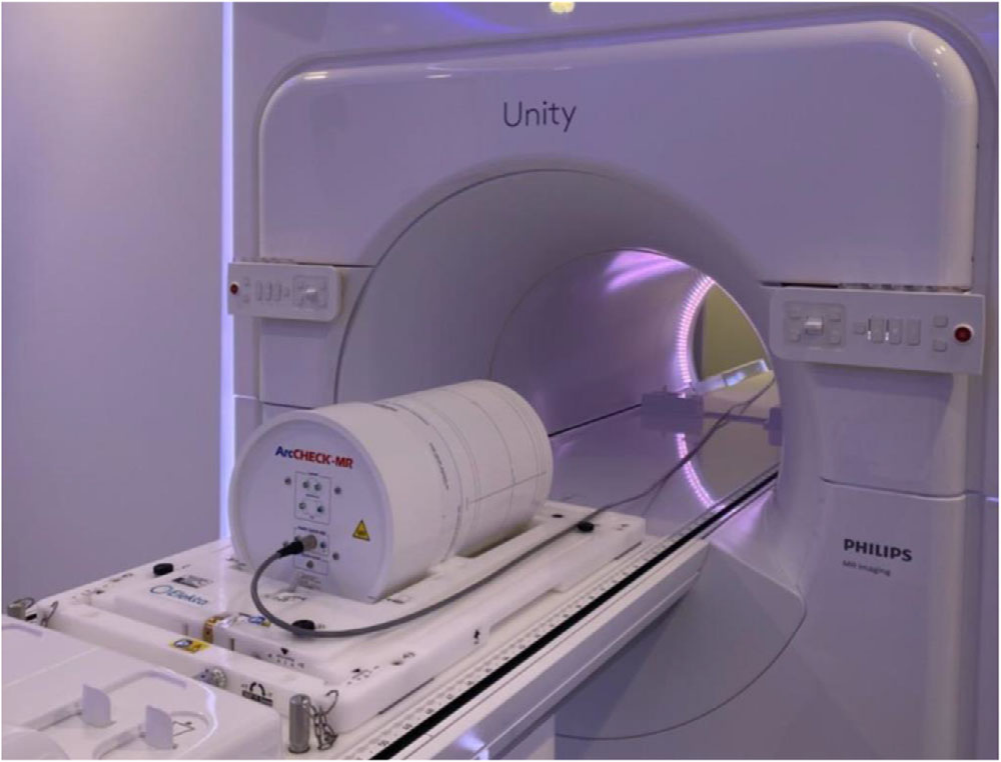
Setup for IMRT QA using the ArcCHECK‐MR diode array positioned on the QA platform.

The reference IMRT plans for the first 50 patients treated with the Elekta Unity were delivered to an MR‐compatible 3D diode array (ArcCHECK‐MR, Sun Nuclear Corporation, Melbourne, FL) as part of patient‐specific quality assurance (PSQA). The treatment sites included pancreas, prostate, and rectum. The plans consisted of 12–16 IMRT beams spaced approximately equidistantly while avoiding hardware structures in the cryostat pipe and couch. PSQA measurements were performed separately for each treatment beam and for the composite plan. The expected and measured dose distributions were compared using gamma criteria of 3% dose difference and 2.0 mm distance‐to‐agreement, global normalization, and 10% low‐dose threshold. A tolerance level of *γ* > 95%, with an action level of *γ* > 90%, was used to evaluate individual beams and composite plan quality.^15^ Figure 5 shows the setup for the ArcCHECK‐MR device on the QA platform.

## 3 RESULTS

### 3.1 MR‐to‐MV concordance and radiation isocenter

**FIGURE 6:**
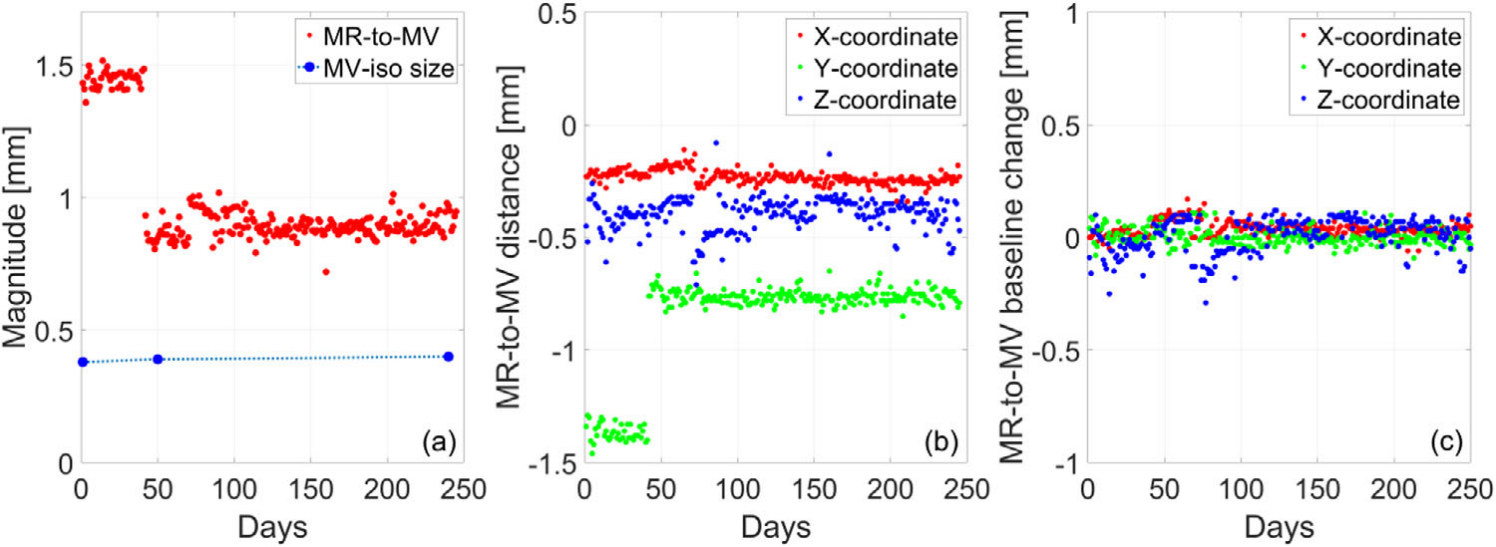
(a) Longitudinal trend of the magnitude of the MR‐to‐MV distance and the size of the MV isocenter. (b) Distance between the MR and MV isocenter along each measurement axis: X = Lt‐Rt, Y = Sup‐Inf, Z = Ant‐Post. (c) Change of MR‐to‐MV distance with respect to baseline along each measurement axis

Figure 6 shows the longitudinal trend of the size of the radiation isocenter and the MR‐to‐MV distance. At commissioning, the baseline values for the MR‐to‐MV distance were –0.23 mm, –1.38 mm, and –0.36 mm in the *X* (Lt‐Rt), *Y* (Sup‐Inf), and *Z* (Ant‐Post) axes, respectively. The baseline was reset and remeasured after machine service and the updated values, also represented by the discontinuity in Figure 6a, were –0.28 mm, –0.65 mm, and –0.42 mm in the *X*, *Y*, and *Z* axes, respectively. After adjustment, the MR‐to‐MV distance was (*μ* ± *σ*) 0.89 ± 0.05 mm. Over a period of 12 months, the change with respect to baseline was (*μ* ± *σ*) 0.039 ± 0.033 mm, 0.0024 ± 0.038 mm, and 0.0068 ± 0.078 mm in the *X*, *Y*, and *Z* axes, respectively.

Based on the Winston–Lutz test, the diameter of the radiation isocenter was 0.39 ± 0.01 mm. Note that MV images at gantry angles of 90° and 270° were consistently excluded from analysis as they were often found to fail field edge detection. This is caused by attenuation in the mounting stand of the MV alignment phantom. The small size of the radiation isocenter is attributed to the design of the ring‐mounted accelerator with tight mechanical tolerances.

### 3.2 MLC and jaw position

**FIGURE 7:**
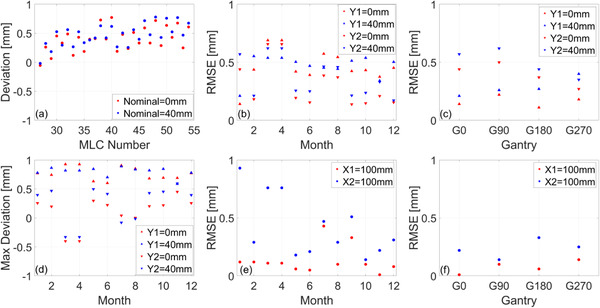
Trend analysis of MLC and jaw positional accuracy. (a) Example deviation from central 28 leaves at two nominal positions. (b) Longitudinal RMSE for each MLC bank at two nominal positions. (c) Gantry dependence of RMSE for each MLC bank measured during commissioning. (d) Longitudinal maximum deviation for each MLC bank at two nominal positions. (e) Longitudinal RMSE for each jaw at the nominal position of 100 mm. (f) Gantry dependence of RMSE for each jaw measured during commissioning. Longitudinal measurements were acquired for gantry at 0°. The dependence on gantry angle is tested annually

Figure 7 plots the longitudinal trend of MLC and jaw positional accuracy. An example of the measurement results from a representative month is shown in Figure 7a for the central 28 MLC leaves. This data is used to calculate RMSE and maximum deviation when monitoring the trend over time and as a function of gantry angle. For both nominal positions (0 and 40 mm), we generally observe that the MLCs in the Y1‐bank have larger RMSE and maximum deviation than those in the Y2‐bank, as seen in Figure 7b). When considering gantry dependence, RMSE was larger for the nominal position of 40 mm than for 0 mm, shown in Figure 7c. A similar behavior is observed in the positional accuracy of the jaws for which the X2 jaw generally has a larger RMSE than the X1 jaw. This was found to also be valid when considering the effect of gantry rotation on jaw positional accuracy. While all measurements are within tolerance, note that jaw and MLC positions are calibrated during periodic maintenance and if a trend toward failure is observed.

### 3.3 Dosimetry of dynamic MLC delivery

**FIGURE 8:**
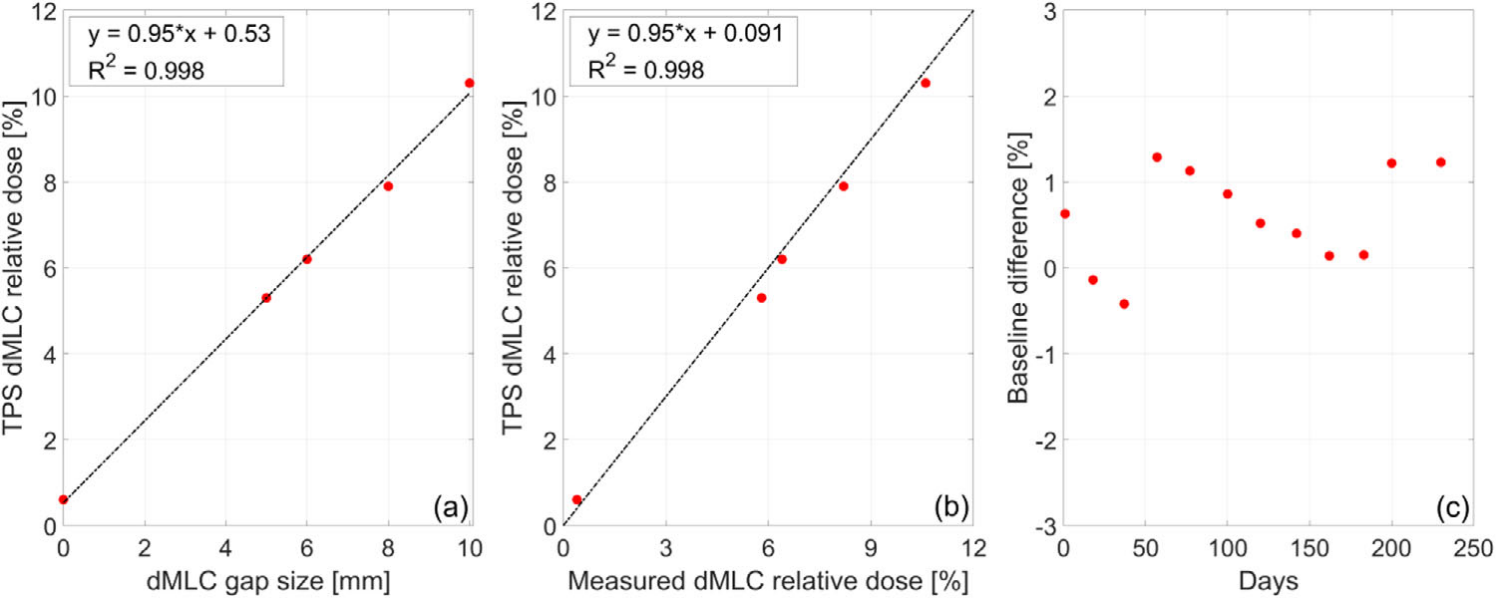
(a) Calculated dMLC relative dose (dMLC output/open field output) as a function of the nominal dMLC gap size. Dashed line shows least‐squares fit. (b) Calculated dMLC relative dose as a function of measured dMLC relative dose. Dashed line shows the identity line. (c) Longitudinal trend of baseline changes in dMLC relative dose for a nominal gap of 10 mm

Figure 8 presents a comparison between calculated and measured dMLC relative dose and the longitudinal trend of baseline changes for monthly measurements. Regression analysis of TPS dMLC relative dose as a function of nominal dMLC gap size shows a strong linear relationship with a coefficient of determination *R* ^2^ = 0.998, as seen in Figure 8a. Using the linear fit estimators, for a 10 mm dMLC gap, a change of 3% in dMLC relative dose corresponds to a dMLC gap change of 0.3 mm. Regression analysis of TPS dMLC relative dose as a function of measured dMLC relative dose shows a strong linear relationship with *R* ^2^ = 0.998, as seen in Figure 8b. The effect of gantry rotation on dMLC relative dose is given in Figure A1 in the Appendix.

### 3.4 Output and beam profile constancy

**FIGURE 9:**
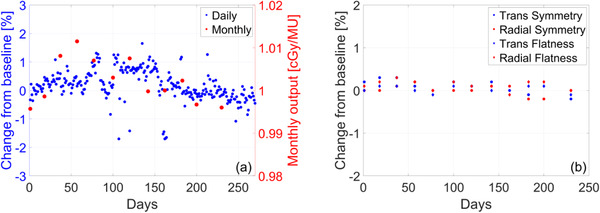
Longitudinal trend of (a) daily output measured with the DQA3‐MR device and monthly output measured in water‐equivalent plastic using the TRS‐483 protocol (b) monthly measurements of radial/transverse symmetry and flatness using an ion‐chamber array

The longitudinal trend of radiation output and beam profile constancy is shown in Figure 9. Output adjustments were made twice during the 12‐month period. Baseline changes for the radial and transverse symmetry were (*μ* ± *σ*) 0.0083% ± 0.12% and 0.075% ± 0.18%, respectively. Baseline changes for the radial and transverse flatness were (*μ* ± *σ*) 0.125% ± 0.11% and 0.025% ± 0.087%, respectively. No adjustments were made to symmetry and flatness during the 12‐month period. A longitudinal trend of output measured daily with the MV portal imager and weekly with the IC Profiler is shown in Figure A2 in the Appendix.

### 3.5 End‐to‐end QA

#### 3.5.1 Point dose comparison: ATP and ATS

**FIGURE 10:**
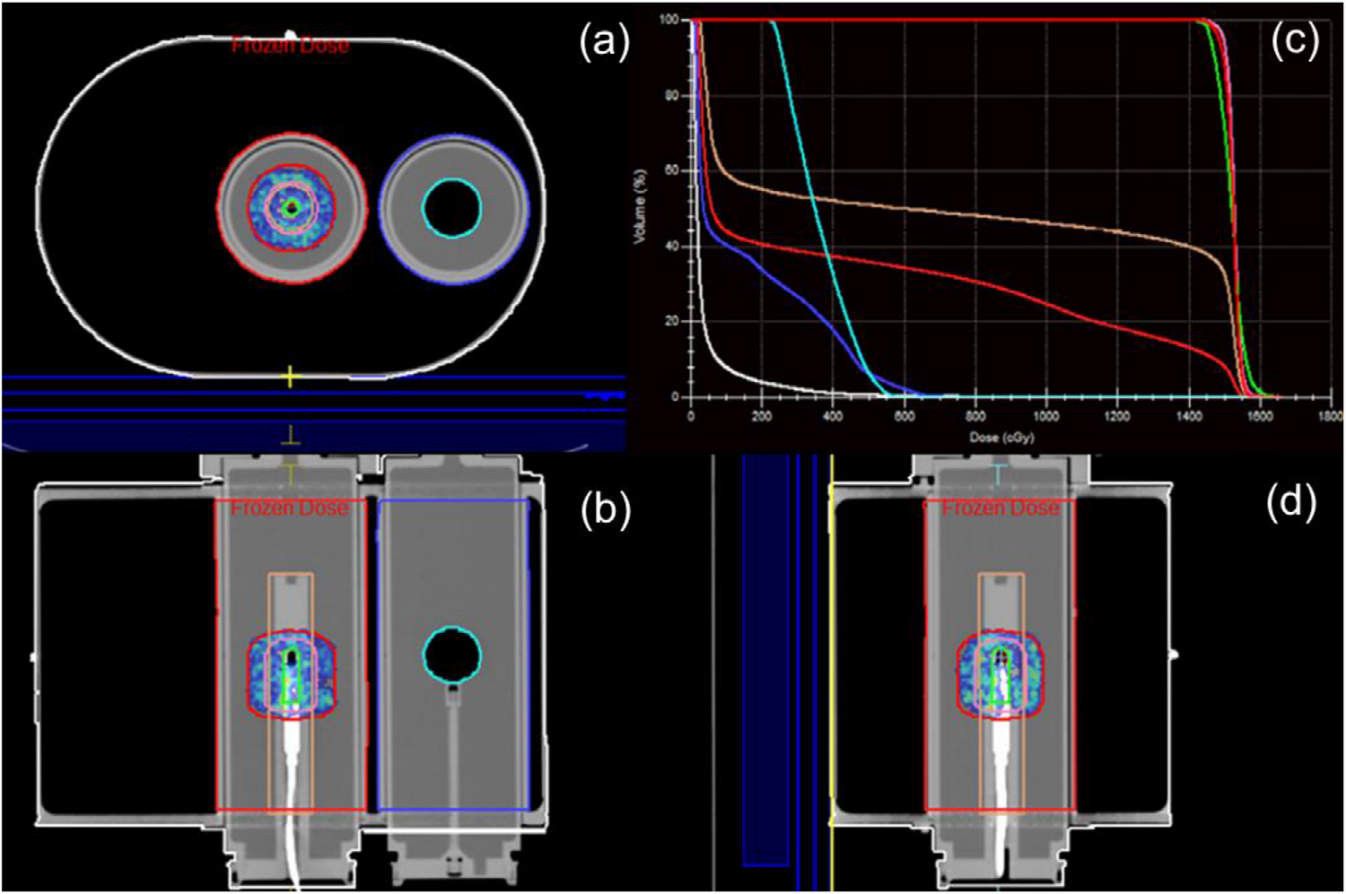
Reference plan dose distribution in the cardinal planes centered on the active volume of the ion‐chamber and dose–volume histogram for all contoured structures. This phantom was found to be particularly helpful in understanding the importance of electron‐density assignment for online plan adaptation

The Quasar MRI4D phantom was used to compare the dose at the ion‐chamber calculated in the treatment planning system with the dose at the ion‐chamber measured after delivery of either ATP or ATS plan adaptation. Figure 10 displays the dose distribution from the reference plan and the dose–volume histogram for all contoured structures. Bulk electron densities were assigned based on the mean Hounsfield units in planning CT. Table 1 shows the results for delivery with plan adaptation using ATP with optimized weights and ATS with optimized weights/shapes from fluence. For ATP, the point dose difference between the treatment planning system and measurement was found to be –1.51%. For ATS, the point dose difference between the treatment planning system and measurement was found to be –0.34%.

**TABLE 1:** Calibration measurements, delivered charge, and dose comparison for online plan adaptation using ATP with optimized weights and ATS with optimizes weights/shapes from fluence

Calibration 10 × 10, 200 MU			Adapt‐to‐position		Comparison	
TPS (cGy)	Charge (nC)	Cal Factor (cGy/nC)	TPS (cGy)	Charge (nC)	Delivered (cGy)	Difference (%)
187.40	–7.87	–23.81	1526.70	–65.09	1549.79	–1.51

			Adapt‐to‐shape		Comparison	
			TPS (cGy)	Charge (nC)	Delivered (cGy)	Difference (%)
			1594.60	–67.20	1600.03	–0.34

#### 3.5.2 Comparison of dose distribution: localization error and dose difference

**FIGURE 11:**
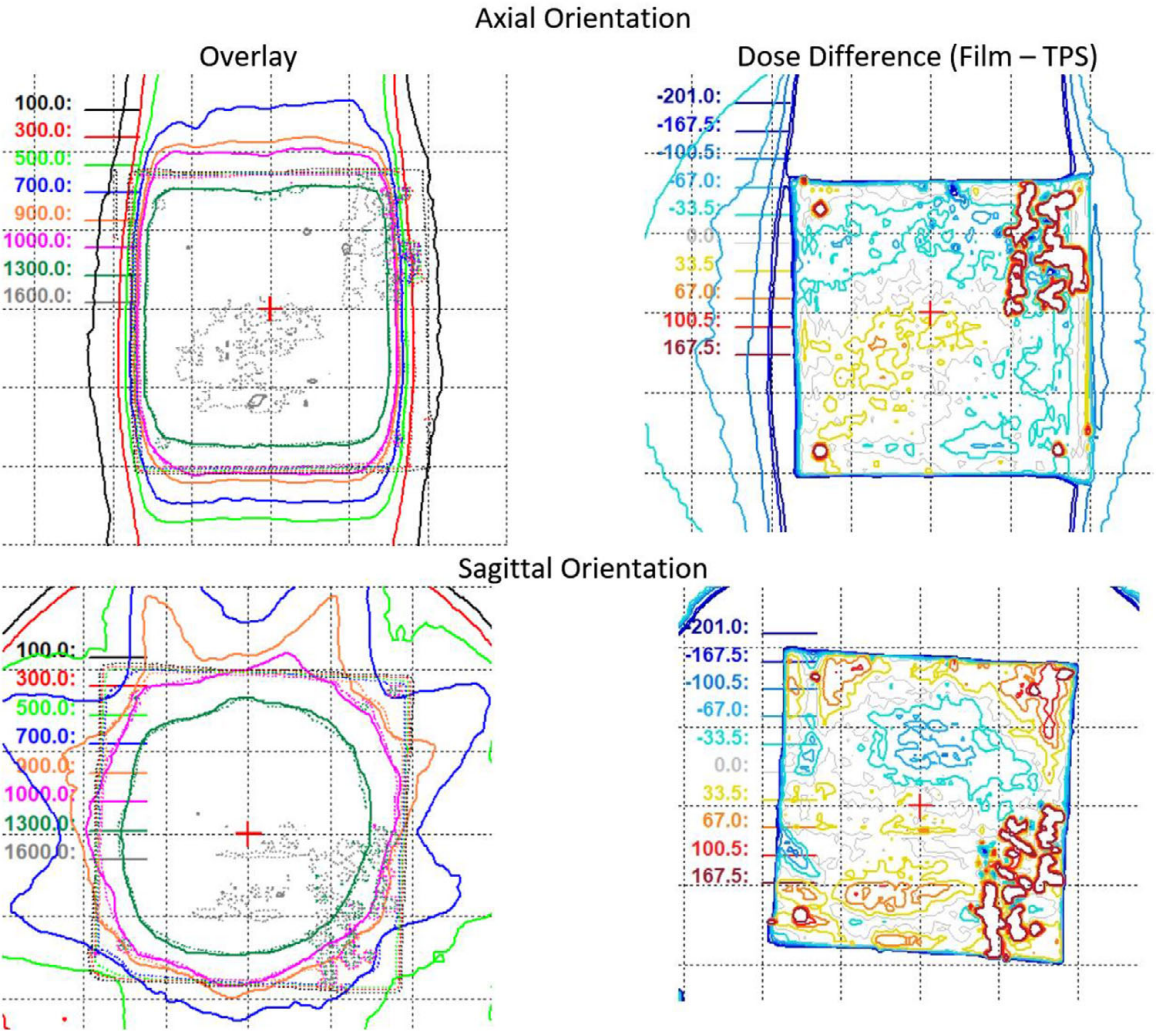
Comparison of calculated (solid lines) and measured (dotted lines) dose for films in axial and sagittal orientation. Overlay of calculated and measured isodose lines (left column) and their respective differences (right column).

The StereoPhan phantom was used to compare calculated dose distribution from an adapted plan and measurement with a radiochromic film placed in the axial and sagittal plane. The treatment plan was delivered twice after adaptation using ATS with optimized weights/shapes from fluence. The analysis was performed in an ROI defined as the 50% of the maximum dose in the plan calculated in the TPS. A comparison of the calculated and measured dose distribution in both planes is shown in Figure 11. The dose difference and localization accuracy were evaluated using tolerance levels of 5% and 1.0 mm, respectively. The results of the analysis for both planes are presented in Table 2.

**TABLE 2:** Average dose difference and localization error in the axial and sagittal plane using radiochromic film measurements with the StereoPhan phantom

	Average dose difference		Localization error [cm]	
	Film‐TPS [cGy]	Film‐TPS [%]	Lt‐Rt	Ant‐Post
**Axial**	4.5	0.3	–0.07	0.09
			Ant‐Post	Sup‐Inf
**Sagittal**	–7.9	–0.5	–0.03	0.07

### 3.6 Patient‐specific QA using MR‐compatible 3D diode array

**FIGURE 12:**
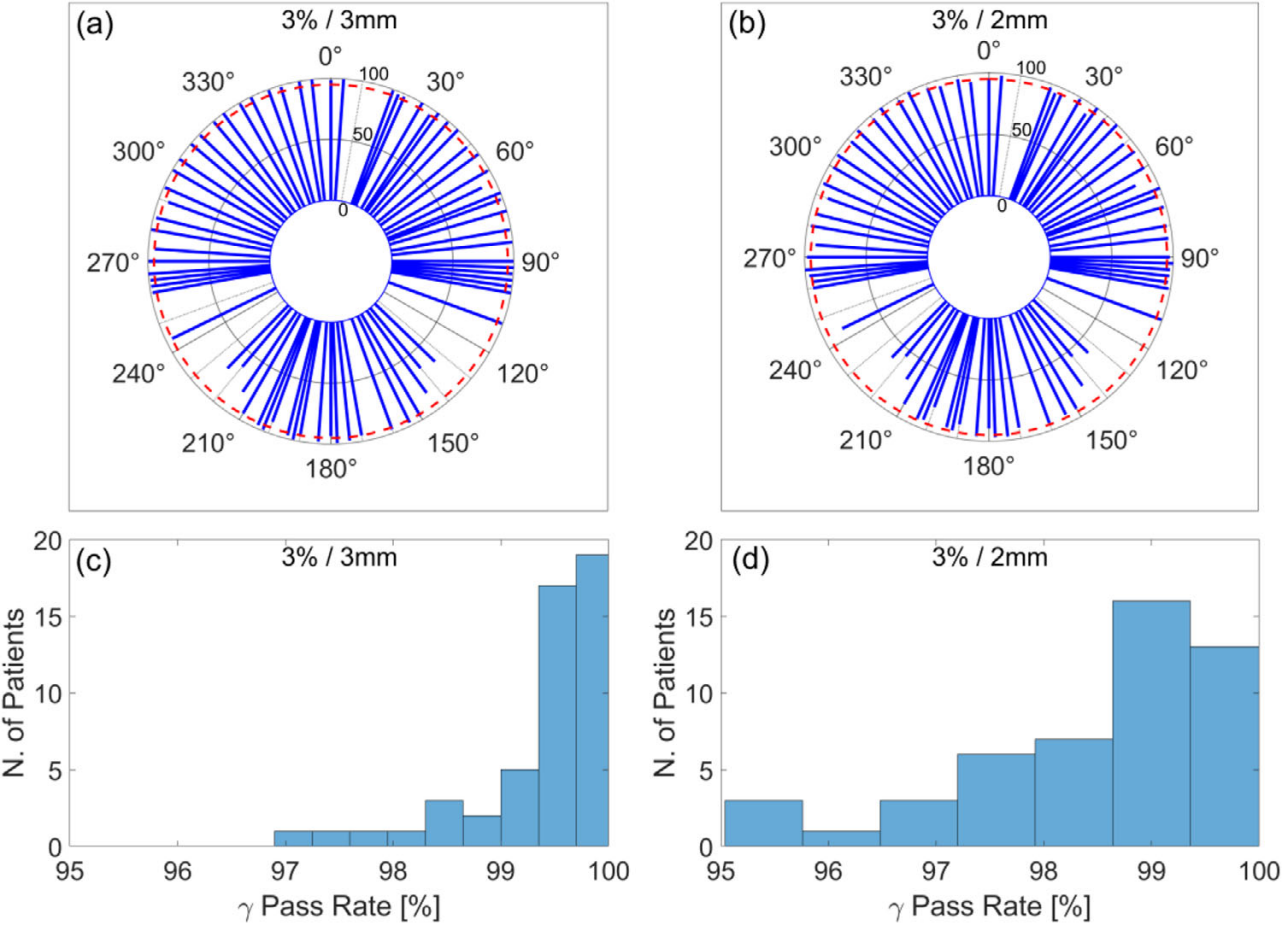
(a), (b) Polar plot of mean gamma passing rate as a function of gantry angle in the reference plans of first 50 patients treated with Unity. Red dashed line indicates gamma passing rate of 95%. (c), (d) Histogram of gamma passing rate for composite plans after adjusting gantry angles that failed IMRT QA.

Figure 12 presents the analysis of IMRT QA measurements for the reference plans of the first 50 patients treated in Unity. Reference and measured plans were compared on a beam‐by‐beam basis and as a composite plan. The histogram of gamma passing rates as a function of gantry angle is shown in Figure 12a,b) for gamma criteria of 3%/3 mm and 3%/2 mm, respectively. Beams that were found to fail IMRT QA were adjusted until passing criteria were met. Figure 12c,d show histograms of passing rates for composite plans using gamma criteria of 3%/3 mm and 3%/2 mm, respectively. Note that the sector of gantry angles for which the beam is attenuated by the couch high‐density material is dependent on the left‐right location of the target. Adaptation for all patients included in this analysis was with ATS and with optimized weights/shapes from fluence.

## 4 DISCUSSION

This report presents the 1‐year longitudinal trend of QA measurements for one of the earliest Elekta Unity MR‐linacs in clinical use in the United States. The focus of this work is on the mechanical and dosimetric performance and stability of the linac component of the machine. The analysis of the QA measurements for the MRI component will be described in a forthcoming publication.

The MR‐to‐MV isocenter concordance is of particular importance in Unity given its use in the online TPS for daily plan adaptation. Figure 6a plots the Euclidian MR‐to‐MV distance and compares it to the size of the radiation isocenter. In our machine, we find the MR‐to‐MV distance to be approximately twice the size of the radiation isocenter, necessitating its use as an input for online plan adaptation. The largest component in the magnitude of the MR‐to‐MV distance was the *Y*‐coordinate which is affected by small variations in the focal spot and waveguide replacement. After machine service for waveguide replacement and realignment, the discrepancy in the Y‐coordinate was decreased by approximately 40%. While during installation the aim is to minimize this discrepancy, there is no recommended tolerance for the distance between the MR‐to‐MV isocenter. At the completion of commissioning, it was decided that the change from baseline would be monitored with a tolerance of 1.0 mm. The recent consortium publication^25^ recommends a tolerance of 0.5 mm from baseline which is supported by our trend analysis.

The longitudinal accuracy of the position of the MLC leaves and jaws is shown in Figure 7. The semiautomated method provided by Elekta determines the position of the central 28 MLC leaves and recommends a tolerance of 1.0 mm RMSE with respect to nominal. The analysis of the maximum error for MLCs reveals that no single leaf deviated by more than 1.0 mm from the nominal over the period of 12 months, as seen in Figure 7d. The consortium recommends a tolerance of 1.0 mm for the position of the MLCs and jaws which is supported by our data. All measurements were within tolerance. The main limitation in this method is related to the constrained FOV in the MV imager (21.0 × 8.5 cm^2^), allowing for QA measurements for only the central 28 leaves. The use of an ion‐chamber array can complement this test and we present our findings with the IC‐Profiler in Figure A3 in the Appendix. Note that the baseline of the expected MLC and jaw position needs to be determined in the TPS as it will be different from the nominal.

Quality assurance tests for the dosimetry of dynamic MLC delivery extend the QA tests for positional accuracy. In particular, dMLC relative dose provides a highly sensitive measure for changes in the position of the MLCs during delivery. Over the period of 1 year, baseline deviations in the dMLC gap width were within 0.3 mm, as seen in Figure 8 and Figure A1 in the Appendix. The longitudinal analysis of baseline changes in monthly measurements indicates a reproducible dosimetry for step‐and‐shoot delivery, which in turn confirms the aggregate reproducibility of dMLC transmission, mechanical integrity, and positional accuracy.

Radiation output was initially cross‐checked daily with the MV panel (at gantry = 0°, 90°), daily with the DQA3‐MR device, weekly with the IC‐Profiler, and monthly with a Farmer‐type ion‐chamber inserted in a water‐equivalent plastic with correction factors from TRS‐483. Over the first 2 months of clinical use, daily output was found to vary within 1% from baseline while weekly measurements were within 0.5% from baseline. No output adjustments were made during this time. After this initial period, daily output was measured with the DQA3‐MR device and monthly with the method described above. Beam profile flatness and symmetry were measured monthly with the IC‐Profiler and found to be within 0.1% and 0.3%, respectively. The baseline of the expected flatness and symmetry was cross‐checked with the TPS.

End‐to‐end testing is conventionally performed during machine commissioning and when implementing new delivery methods or treatment sites. While typically not tracked longitudinally, the measurements determine the uncertainty of the entire treatment chain and set a baseline for subsequent monitoring. In an MR‐linac, particular attention is given to the use of an anthropomorphic phantom with materials visible both in CT/MR and detectors with minimal response to the effects of the magnetic field. The community is still in need of anthropomorphic phantoms optimized for end‐to‐end testing in the MR‐Linac. While ion‐chamber response is well understood in the presence of the magnetic field, it provides measurements only for point dose comparisons. The inclusion of film measurements necessitates the use of phantoms that minimize any air gaps in the holder while also providing sufficient CT and MR signal for planning and online adaptation. We find that phantoms with large heterogeneities are especially useful in understanding the features of online image registration (rigid and deformable) and the uncertainties arising from electron‐density assignments for online plan adaptation.

Patient‐specific QA for the first 50 cases was performed for composite plans and beam‐by‐beam. We find that beams passing through high‐density structures in the couch are likely to have low‐pass rates in IMRT QA and may need adjustments during online planning. This is due to the representation of the couch model in the TPS and the voxel size chosen for dose calculation. A common solution in our workflow was to adjust the gantry angle by 5°–10° in a direction away from the highly attenuating structures. This change provided a passing rate for IMRT QA for the specific beam and overall improvement in composite plan gamma passing rate. Note that the sector of gantry angles for which the beam is attenuated by the couch high‐density material is dependent on the left‐right location of the target. Some treatment beams were found to fail IMRT QA even when it was determined from the beam's eye‐view that the couch structures were not attenuating. Beam adjustments led to subsequent passes in all fractions after online adaptation.

## 5 CONCLUSION

We report the 1‐year longitudinal trend of linac QA measurements for an Elekta Unity machine in clinical use in our institution. Our findings show that the device operates within the guidelines of current recommendations for linear accelerator performance, stability, and safety. The analysis of the data supports the recently published guidance in establishing clinically acceptable tolerance levels for relative and absolute measurements.

## CONFLICT OF INTEREST

The authors declare that there is no conflict of interest.

## AUTHOR CONTRIBUTIONS

All authors contributed to the conception and design of the work, drafting, and final approval of manuscript.

## Supporting information

Appendix
