# Supplementary material for: Longitudinal assessment of quality assurance measurements in a 1.5T MR‐linac: Part I—Linear accelerator
Source: J Appl Clin Med Phys. 2021 Sep 10;22(10):190–201. doi: 10.1002/acm2.13418 (PMC8504604; doi:10.1002/acm2.13418)
Supplement: Supplementary file 1 — Appendix [file ACM2-22-190-s001.docx]

**APPENDIX**


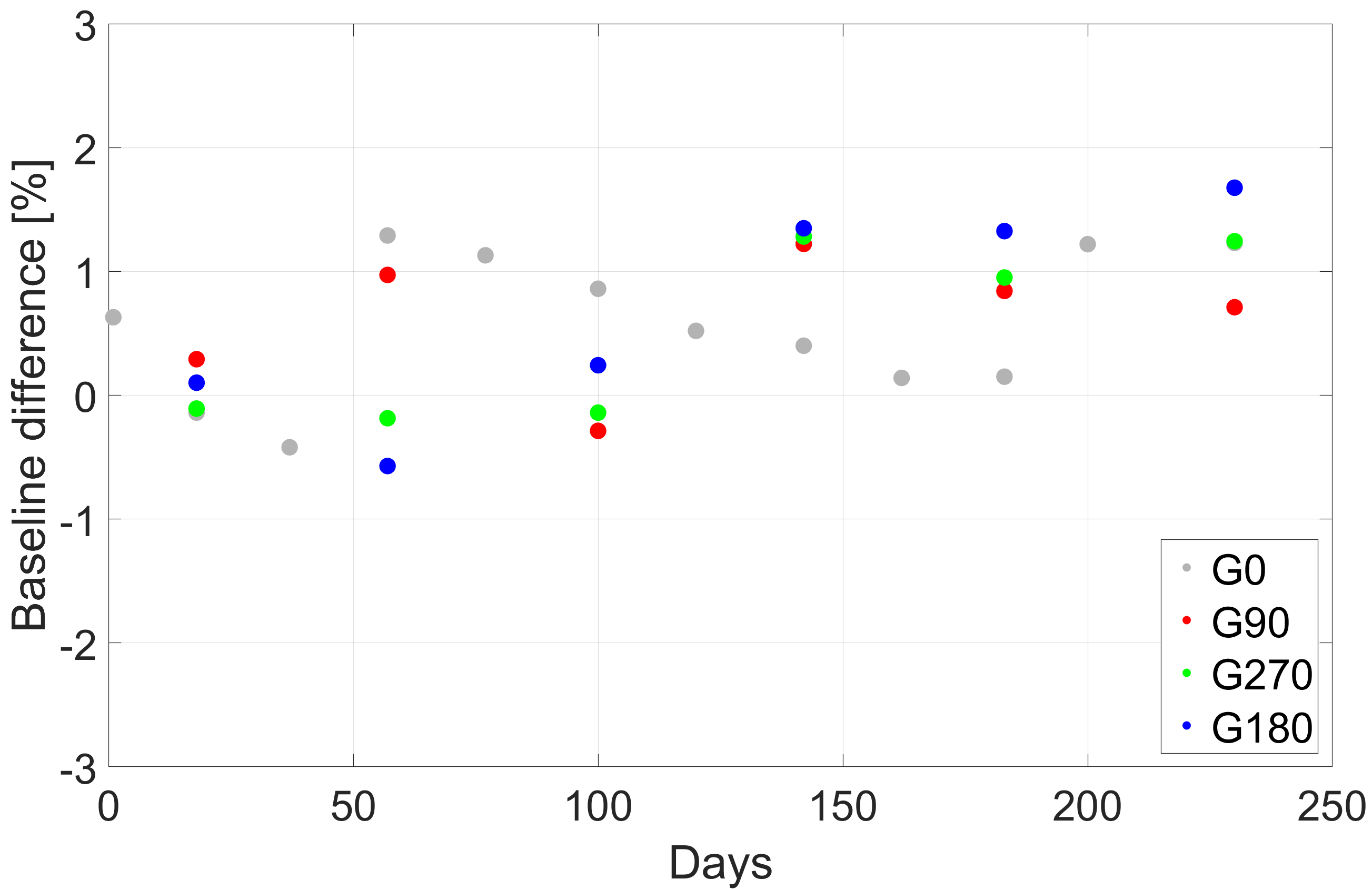


Figure A1. Longitudinal trend of baseline changes in dMLC relative dose for a nominal gap of 10 mm at the cardinal gantry angles.


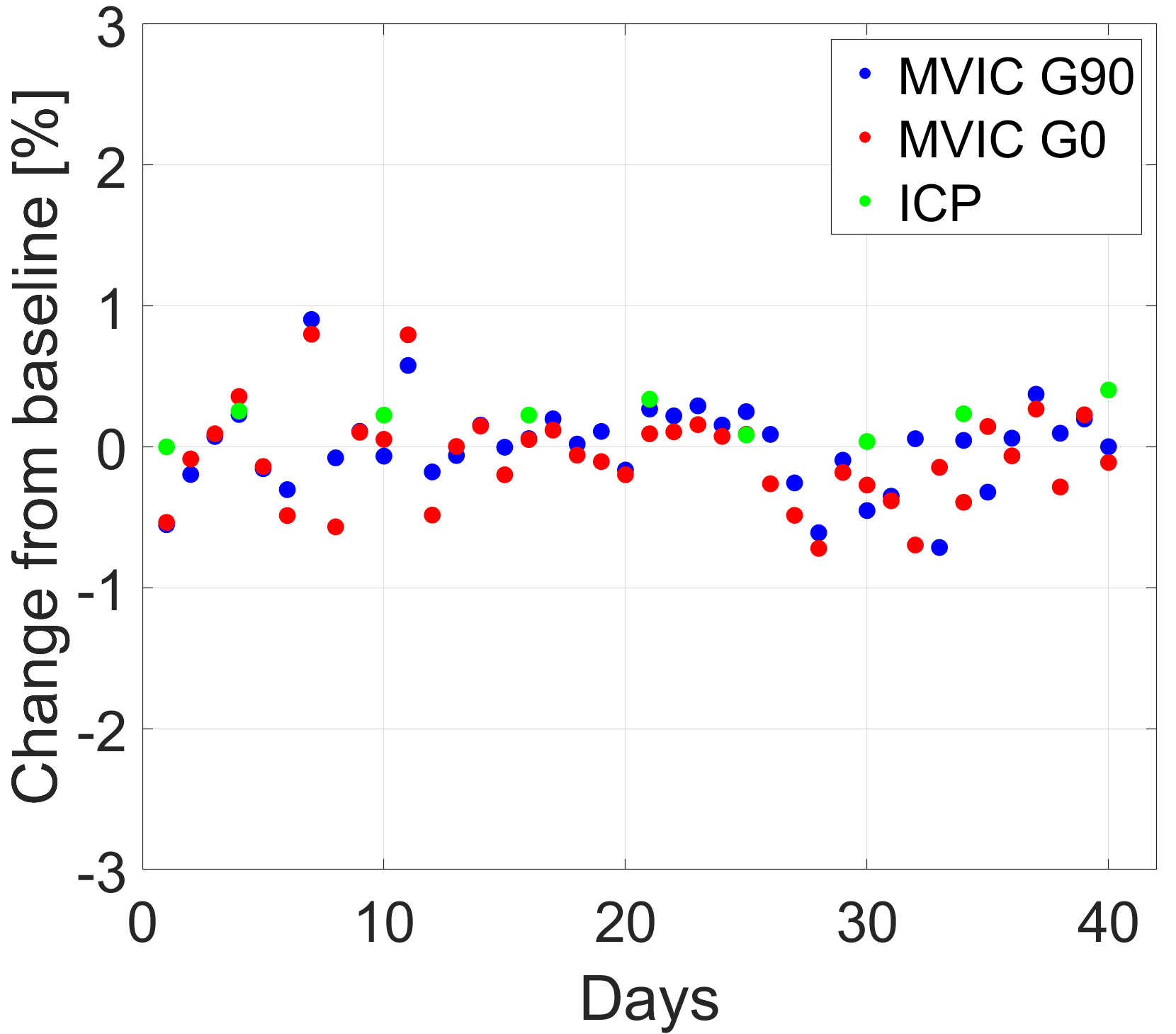


Figure A2. Longitudinal trend of daily output measured with the MV portal imager (MVIC) and weekly output measured with an ion-chamber array.


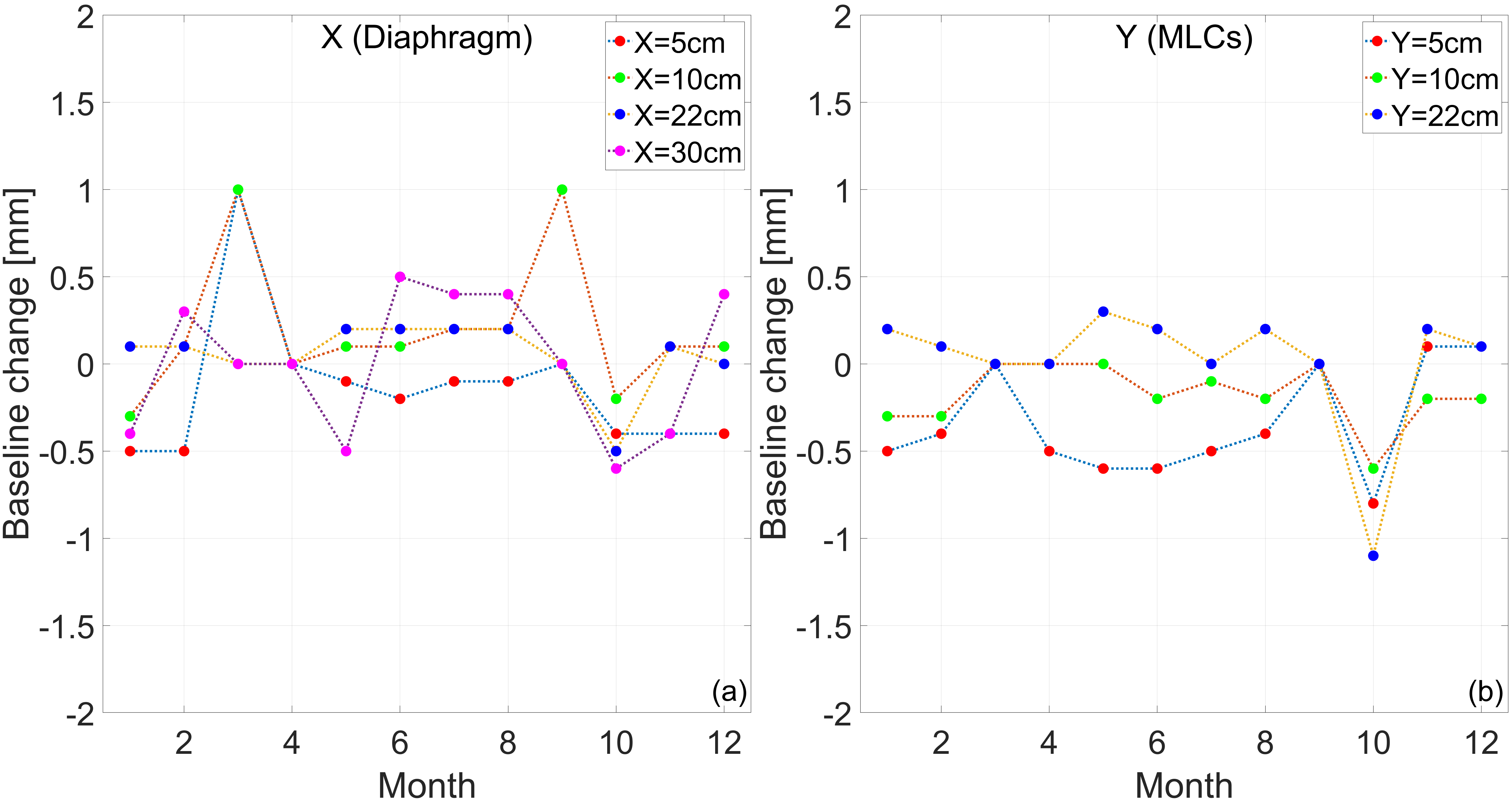


Figure A3. Trend analysis of MLC and jaw positional accuracy measured with an ion-chamber array. (a) Longitudinal changes with respect to baseline for diagram. (b) Longitudinal changes with respect to baseline for MLCs.
